# The Correlation between the Change in Thoracic Fluid Content and the Change in Patient Body Weight in Fontan Procedure

**DOI:** 10.1155/2018/3635708

**Published:** 2018-05-09

**Authors:** Tae-Gyoon Yoon, Kyunghwan Jang, Chung-Sik Oh, Seong-Hyop Kim, Woon-Seok Kang

**Affiliations:** ^1^Department of Anesthesiology and Pain Medicine, Sejong Hospital, Bucheon, Gyeonggi-do, Republic of Korea; ^2^Department of Anesthesiology and Pain Medicine, Konkuk University Medical Center, Konkuk University School of Medicine, Seoul, Republic of Korea; ^3^Institute of Biomedical Science and Technology, Konkuk University School of Medicine, Seoul, Republic of Korea

## Abstract

**Background:**

The thoracic fluid content (TFC) and its percent change compared to the baseline (TFCd0%) derived from a bioreactance technique using a noninvasive cardiac output monitoring (NICOM) device correlate well with the amount of fluid removal in patients undergoing hemodialysis and with intraoperative fluid balance in pediatric patients undergoing cardiac surgery. We hypothesized that TFC or TFCd0% would also be a useful indicator allowing fluid management in pediatric patients undergoing a Fontan procedure.

**Methods:**

The medical records of patients who underwent an elective Fontan procedure were reviewed retrospectively. The intraoperative variables recorded at two time points were used in the analysis: when the NICOM data obtained just after anesthesia induction (*T*0) and just before transfer of the patient from the operating room to the ICU (*T*1). The analyzed variables were hemodynamic parameters, TFC, TFCd0%, stroke volume variation, body weight gain, change in the central venous pressure, and difference in the TFC (ΔTFC).

**Results:**

The correlation coefficient between TFCd0% and body weight gain was 0.546 (*p* = 0.01); between TFCd0% and body weight gain% 0.572 (*p* = 0.007); and between TFCd0% and intraoperative fluid balance 0.554 (*p* = 0.009). The coefficient of determination derived from a linear regression analysis of TFCd0% versus body weight gain was 0.30 (*p* = 0.01); between TFCd0% and body weight gain% 0.33 (*p* = 0.007); and between TFCd0% and intraoperative fluid balance 0.31 (*p* = 0.009).

**Conclusions:**

TFCd0% correlated well with body weight gain, body weight gain%, and intraoperative fluid balance. It is a useful indicator in the intraoperative fluid management of pediatric patients undergoing a Fontan procedure.

**Trial Registration:**

This trial is registered with Clinical Research Information Service KCT0002062.

## 1. Introduction

During pediatric cardiac surgery, an accurate determination of the patient's volume status, and the choice of an appropriate fluid management strategy are important factors leading to a stable hemodynamic status and thus a better clinical outcome. These considerations are even more important than in adult cardiac surgery, due to the potential for large fluid shifts compared with the pediatric patient's small body surface area [1–3]. In addition, inflammatory reactions induced by cardiopulmonary bypass (CPB) can aggravate an unstable hemodynamic state [4].

In adult patients, volume status is traditionally evaluated by measuring the central venous pressure (CVP) or pulmonary capillary wedge pressure (PCWP) in the past. It is now widely demonstrated that cardiac filling pressures could not reliably predict fluid responsiveness. CVP also has been widely used to estimate the intravascular volume status of pediatric patients because of any other suitable alternative parameter for fluid status. However, as an indicator of exact volume status its use in this population is inappropriate, due to various factors that affect its accuracy, such as venous capacitance, cardiac chamber compliance, cardiac valve competence, and pulmonary artery pressure [5–7]. Measurement of PCWP in pediatric patients with congenital heart disease is technically challenging, as the patient's small size or an aberrant anatomy makes catheter insertion into the pulmonary artery difficult. Moreover, this procedure has a high risk of complications [8, 9].

In pediatric patients undergoing a Fontan procedure, the exact volume status cannot be determined based on the CVP value, because the latter is actually the postoperative pulmonary arterial pressure following bidirectional cavopulmonary shunt (BCPS). The development of a good indicator of the volume status of pediatric patients will allow fluid management in those undergoing a Fontan procedure.

The thoracic fluid content (TFC) and its percent change compared to the baseline (TFCd0%) are derived from the application of a bioreactance technique performed using a noninvasive cardiac output monitoring (NICOM) device. The values obtained with this method correlate well with the amount of fluid removal in patients undergoing hemodialysis [10] and with intraoperative fluid balance in pediatric patients undergoing cardiac surgery [11]. We therefore hypothesized that TFC or TFCd0% would also be a useful indicator allowing fluid management in pediatric patients undergoing a Fontan procedure. We explored the utility of this approach by analyzing potential correlations between TFC, TFCd0%, intraoperative fluid balance, and changes in patient body weight.

## 2. Methods

### 2.1. Clinical Data

This study was approved by the Institutional Review Board of Konkuk University Medical Center, Seoul, South Korea [KUH1160103, (Aug, 2016)], and subsequently registered at http://cris.nih.go.kr (KCT0002062). The medical records, including the anesthetic and operative records, of patients with a congenital functional single ventricle who underwent an elective Fontan procedure at Konkuk University Medical Center from April 2011 to December 2015, were reviewed retrospectively. Demographic, diagnostic, procedural, and hemodynamic information were obtained and analyzed.

Anesthesia induction and maintenance were performed according to the institutional standard regimen for pediatric cardiac surgery: sufentanil-based anesthesia with sevoflurane. Anesthesia induction was followed by invasive arterial pressure monitoring at radial artery and central venous catheterization for CVP monitoring at internal jugular vein. Mean arterial pressure (MAP) and CVP were measured using pressure transducers (PX600F, Edwards Lifesciences, Irvine, CA, USA), which were placed on the patient at the level of the mid-axillary line under the guidance of a laser leveler (Physiotrac, Edwards Lifesciences) and then fixed to the operation table. Two NICOM electrode strips (right and left) were then placed on each side of the chest of the patient and connected to a NICOM controller (NICOM, Cheetah Medical, Vancouver, WA, USA). Each electrode strip consisted of upper and lower contact points. The upper thoracic electrode strips were placed at the mid-subclavian region and the lower electrode strips in the middle region of the lower costal margin. After an initial calibration of the electrodes, hemodynamic variables, including cardiac output (CO), cardiac index (CI), stroke volume (SV), and stroke volume variation (SVV), were monitored continuously and recorded every 10 min until just before the patient was transported to the intensive care unit (ICU). After surgical procedure was over, the patients were transported to the ICU with intubation status.

The system's signal processing unit determines the relative phase shift (*Ф*) between input and output signals, which reflects changes in blood volume in the aorta. SV determined by NICOM can be estimated using SV = *C* · VET · *dФ*/*dt*_max_, where *C* is a constant of proportionality, VET is ventricular ejection time, and *dФ*/*dt*_max_ is the peak rate of change of Ф [10]. The value of *C* has been optimised in prior studies, accounting for patient age, gender, and body size [11]. Maximal and minimal values of beat-to-beat SV were determined over a single respiratory cycle. SVV was calculated as SVV (%) = (SVVmax − SVVmin)/(SVVmax + SVVmin/2) · 100.

The water content of the body, whether in the blood (intravascular) or outside the blood (extravascular), contains high concentrations of various electrolytes, such as sodium, chloride, potassium, and calcium. These electrolytes are good conductors of electricity. The more fluid a patient has in his/her chest, the more electrolytes are available for electrical conductance and vice versa. Thoracic fluid content (TFC) is calculated as TFC = 1/*Z*_0_, where *Z*_0_ is basic thoracic impedance (unit: Ohms), moving in the opposite direction to the water content. TFC provides a relative measure of changes in thoracic fluid. Specifically, higher TFC values compared with baseline indicate that the water content of the patient's chest is increased and vice versa. TFCd0% is the percentage change in TFC compared with the baseline TFC value at the measured point, and it changes with variations in TFC. Parameters measured by the NICOM device were average values during 1 min, with a 1 min interval between measurements.

Among the intraoperative variables, those recorded at two time points were used in the analysis: the NICOM data obtained just after anesthesia induction (*T*0) and just before transfer of the patient from the operating room to the ICU (*T*1). The analyzed variables were MAP (mmHg), heart rate (HR, beats/min), CVP (mmHg), CO (L/min), CI (L/min/m^2^), TFC (1/Ω), TFCd0% (%), and SVV (%). The body weight gain of the patient, the change in the CVP, and the difference in the TFC (ΔTFC) were calculated as the difference between the values at *T*1 and *T*0. The intraoperative fluid balance (intake/output) was estimated based on intraoperative fluid and blood product administration, urinary output, and the fluid balance during CPB.

Cardiac surgery procedures were performed by a single pediatric cardiac surgery team. CPB was performed by a single perfusionist on that team. All patients included in the study underwent an extracardiac Fontan procedure.

### 2.2. Determination of Patient Body Weight

The patient's body weight was measured twice, according to the standard protocol for pediatric cardiac surgery at our institution. After anesthesia induction with routine invasive (arterial blood pressure) and noninvasive patient (pulse oximetry, electrocardiography) monitoring, a central venous catheter and a urinary catheter were inserted and the electrode strips of the NICOM were attached. The patient's body weight was then measured using portable scales (SW-30H, CAS, Yang-ju, Republic of Korea). This was the *T*0 value. Just before the patient was moved from the operating room to the ICU, his or her body weight was measured again (*T*1) using the same method. The percent gain in body weight was calculated as follows: body weight gain% = 100 × (body weight at *T*1 − body weight at *T*0)/body weight at *T*0.

### 2.3. Statistics

Statistical analyses were conducted using SigmaStat software (ver. 3.1; SYSTAT Software, San Jose, CA, USA). Continuous variables were analyzed using the paired *t*-test or Wilcoxon's signed-rank test, to compare the values obtained at the two measurement points. Student's *t*-test or the Mann–Whitney rank sum test was used to compare values between groups. Categorical variables were analyzed using a *χ*^2^ test. Correlations between variables were determined using the Pearson product moment correlation analysis and linear regression analysis. The data are expressed as the mean ± standard deviation or as the median (25th to 75th percentile) and the number of patients. A *p* value < 0.05 was considered to indicate statistical significance.

## 3. Results

From April 2011 to December 2015, data from the medical records of 22 patients were collected. One patient was excluded for incomplete medical records. The preoperative demographic profiles of the 21 patients included in the study are summarized in Table 1. During the preoperative period, all 21 patients had a BCPS and had already undergone surgery at least twice.

There were no differences in MAP, CO, and CI at *T*1 versus *T*0, whereas HR, CVP, TFC, SVV, and body weight were significantly higher at *T*1 (*p* < 0.001, *p* < 0.001, *p* < 0.001, *p* = 0.009, and *p* < 0.001, resp.; Table 2).

The body weight gain was 690.0 ± 307.1 g, corresponding to a body weight gain% of 5.5 ± 2.2%. Intraoperative fluid balance was 680.4 ± 332.2 mL. The change in the CVP was 3.8 ± 3.3 mmHg; ΔTFC was 14.1 ± 5.5 1/Ω; TFCd0% and SVV at *T*1 were 30.1 ± 13.3% and 15.6 ± 2.9%, respectively (Table 3).

The patients were divided into low and high TFCd0% groups based on a mean TFCd0% value at *T*1 of 30. Body weight gain, body weight gain%, intraoperative fluid balance, the absolute CVP value, the change in the CVP value, and ΔTFC were compared between groups. Among these values, body weight gain, body weight gain%, intraoperative fluid balance, and ΔTFC were significantly higher in the high TFCd0% group than in the low TFCd0% group, whereas neither the absolute value of CVP nor the change in CVP differed between groups (Table 4).

The correlation coefficient between TFCd0% and body weight gain was 0.546 (*p* = 0.01); between TFCd0% and body weight gain% 0.572 (*p* = 0.007); and between TFCd0% and intraoperative fluid balance 0.554 (*p* = 0.009). The correlation coefficient between ΔTFC and body weight gain was 0.449 (*p* = 0.41); between ΔTFC and body weight gain% 0.479 (*p* = 0.028); and between ΔTFC and intraoperative fluid balance 0.518 (*p* = 0.016). The correlation coefficient between the change in the CVP value and body weight gain was 0.494 (*p* = 0.023); between the change in the CVP value and body weight gain% 0.510 (*p* = 0.018); and between the change in the CVP value and intraoperative fluid balance 0.482 (*p* = 0.027). The correlation coefficient between body weight gain and intraoperative fluid balance was 0.821 (*p* < 0.001).

**Figure 1:**
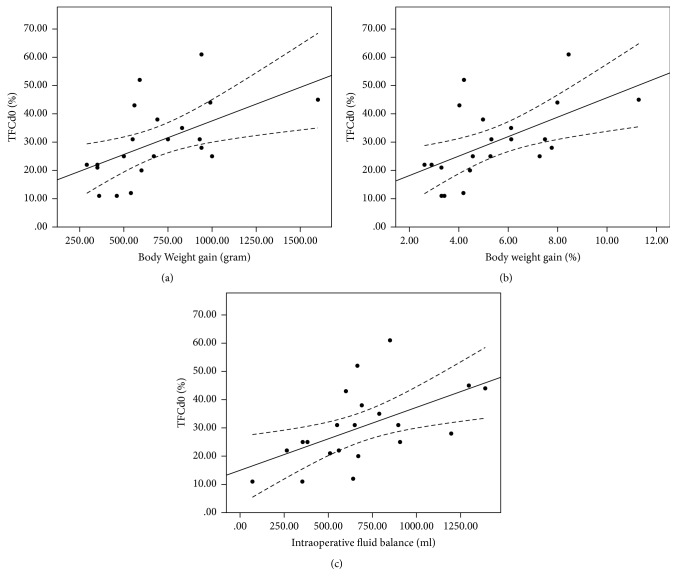
Linear regression analysis between TFCd0% and body weight gain ((a), *R*^2^ 0.30), between TFCd0% and body weight gain% ((b), *R*^2^ 0.33), and between TFCd0% and intraoperative fluid balance ((c), *R*^2^ 0.31).

**Figure 2:**
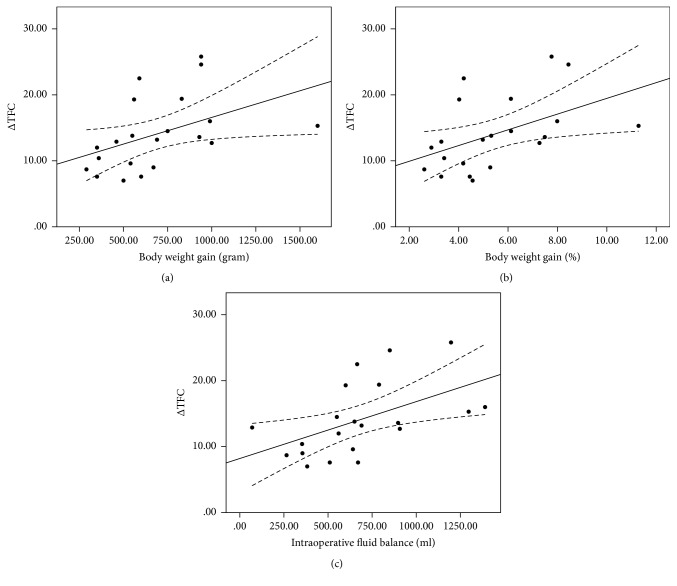
Linear regression analysis between ΔTFC and body weight gain ((a), *R*^2^ 0.20), between ΔTFC and body weight gain% ((b), *R*^2^ 0.23), and between ΔTFC and intraoperative fluid balance ((c), *R*^2^ 0.27).

**Figure 3:**
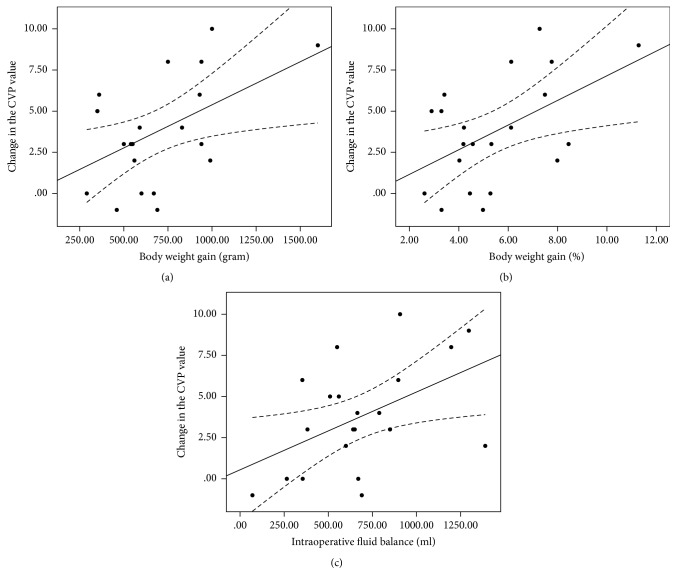
Linear regression analysis between the change in the CVP value and body weight gain ((a), *R*^2^ 0.24), between the change in the CVP value and body weight gain% ((b), *R*^2^ 0.26), and between the change in the CVP value and intraoperative fluid balance ((c), *R*^2^ 0.23). CVP, central venous pressure.

**Figure 4:**
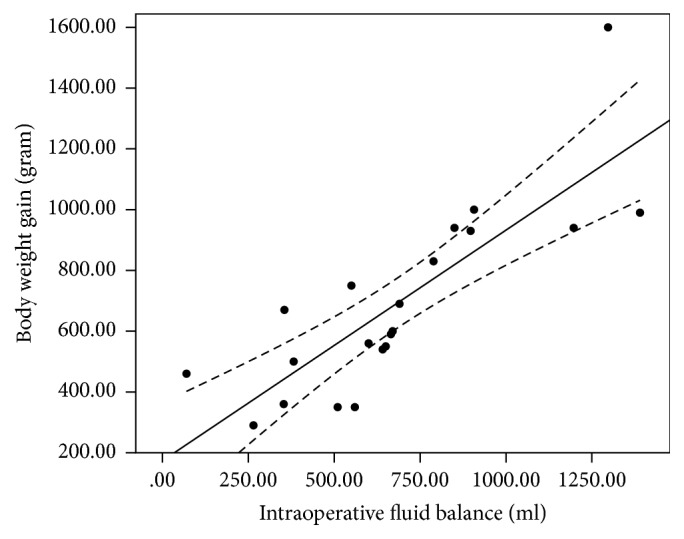
Linear regression analysis between body weight gain and intraoperative fluid balance (*R*^2^ 0.67).

The coefficient of determination (*R*^2^) derived from a linear regression analysis of TFCd0% versus body weight gain was 0.30 (*y* = 13.76 + 0.02*x*, *p* = 0.01); between TFCd0% and body weight gain% 0.33 (*y* = 11.30 + 3.44*x*, *p* = 0.007); and between TFCd0% and intraoperative fluid balance 0.31 (*y* = 14.99 + 0.02*x*, *p* = 0.009) (Figure 1). The *R*^2^ value between ΔTFC and body weight gain was 0.20 (*y* = 8.50 + 0.008*x*, *p* = 0.041); between ΔTFC and body weight gain% 0.23 (*y* = 7.54 + 1.19*x*, *p* = 0.028); and between ΔTFC and intraoperative fluid balance 0.27 (*y* = 8.20 + 0.009*x*, *p* = 0.016) (Figure 2). The *R*^2^ between the change in the CVP value and body weight gain was 0.24 (*y* = 0.15 + 0.005*x*, *p* = 0.023); between the change in the CVP value and body weight gain% 0.26 (*y* = −0.34 + 0.75*x*, *p* = 0.018); and between the change in the CVP value and intraoperative fluid balance 0.23 (*y* = 0.55 + 0.005*x*, *p* = 0.027) (Figure 3). The *R*^2^ value between body weight gain and intraoperative fluid balance was 0.67 (*y* = 177.56 + 0.759*x*, *p* < 0.001) (Figure 4).

## 4. Discussion

In the present study, the values of the correlation coefficients derived from the correlation analyses were acceptable, with the strongest correlations between TFCd0% and body weight gain and between body weight gain% and intraoperative fluid balance. The coefficients of determination derived in the linear regression analysis were also acceptable, with the highest being those between TFCd0% and body weight gain and between body weight gain% and intraoperative fluid balance. Because the intraoperative fluid balance is almost the only factor that affects patients' body weight and the result derived from linear regression analysis between body weight gain and intraoperative fluid balance showed high correlation coefficient, these findings suggest that TFCd0% is an appropriate indicator for intraoperative fluid management in pediatric patients undergoing a Fontan procedure.

In pediatric patients with congenital heart disease who are undergoing surgery, CVP is an inappropriate parameter for estimating the exact volume status. This can be explained by factors such as intracardiac shunt (left to right or right to left), altered pulmonary vascular resistance, altered left ventricular and right ventricular compliance, and the complex anatomy that often occurs in congenital heart disease [7]. Moreover, even after complete correction of the pathology, CVP does not indicate the patient's actual volume status [12]. The results of this study, in which the correlation coefficient and the coefficient of determination of CVP versus TFCd0% were low, corresponded well with those of previous reports.

In comparisons of the variables between *T*0 and *T*1, the values of HR, CVP, TFC, and SVV were higher at *T*1 than at *T*0. The higher HR and SVV values may have been related to postoperative conditions, such as those resulting from the use of inotropes, a lack of effective circulating blood volume, and the inflammatory reaction induced by CPB. The higher TFC and body weight may have been due to a positive intraoperative fluid balance. There were various possible reasons for the higher CVP at *T*1 than at *T*0. It may have been related to more blood volume crossing the pulmonary vascular bed, higher PVR from exposure to cardiopulmonary resistance, and elevated end diastolic pressure due to myocardial edema. However, it is not clear what was the definite reason because the methods for their appropriate measurement of pulmonary blood volume or PVR are lacking.

Prior to undergoing a Fontan procedure, most patients have already been treated surgically and have a postoperative status of BCPS. This was the case in the present study, in which all of the patients had previously undergone a BCPS procedure. Therefore, the CVP value was instead a measurement of the pulmonary artery pressure (PAP), both before and after surgery. In the determination of PAP, pulmonary vascular resistance is the important factor; however, it is also affected by other factors and, thus, does not reflect the exact volume status of the patient.

The patients were divided into two groups based on the value of TFCd0% 30% at *T*1. This allowed an evaluation of the relationship between TFCd0% and other variables. Comparisons between the low and high TFCd0% groups with respect to body weight gain, intraoperative fluid balance, CVP, and the changes in the TFC showed that only the absolute value and the change in CVP did not differ between groups. All other variables were higher in the high TFCd0% group. This demonstrates that the absolute CVP value and the change in CVP do not reflect either the change in the patient's body weight or the intraoperative fluid balance. Instead, both can be determined using the TFCd0%, which may also be an appropriate indicator in intraoperative fluid management.

In a previous study, TFCd0% correlated well with intraoperative fluid balance and with body weight gain, based on correlation coefficients of ~0.7 [11]. Although the values of the correlation coefficients in the present study were acceptable, they were only ~0.5. This may have been due to the longer operation time (220 min versus 160 min in the previous study) and to previous multiple operations. During prolonged surgery, there is a greater redistribution of administered fluid to the interstitial tissue, which progresses to involve the whole body, including the thoracic area. In addition, our patients had already been operated on at least twice previously, such that greater surgical bleeding and therefore a larger fluid shift could be expected compared with first-time surgery patients. Because TFC is calculated by measuring the electrical conductance in the chest [13, 14], it does not take into account the fluid status in the extremities, resulting in a lower correlation coefficient than obtained in a previous study [11].

Among the limitations of the present study was its retrospective design. A prospective study would be able to clarify the relationships between the variables. However, the present study has been performed as retrospective design. Although all the medical records were reviewed carefully, the limitations inherent in a retrospective study were inevitable. In addition, TFCd0% detects the changes in intra- and extravascular volume status based on the electrical conductance in the patient's chest, but it does not distinguish intravascular from extravascular volume change. However, when a large amount of fluid is administered to maintain a stable hemodynamic state, large fluid shift to the third space and tissue edema occurs more often than otherwise. In this case, since TFCd0% reflects the intraoperative fluid balance well, the measurement of TFCd0% can be useful to detect and prevent the side effects of large fluid administration. Thus, in addition to hemodynamic parameters and TFCd0%, the simultaneous use of the dynamic parameters derived from heart–lung interactions, indicative of fluid responsiveness, would improve intraoperative fluid management [15–17].

In the clinical field, fluid balance is difficult to estimate accurately, due to conditions such as continuous surgical bleeding in the operating field, absorption of surgical bleeding by gauzes, and the insensible loss of body fluid. Furthermore, estimates based on the intake and output of intraoperative fluid are time-consuming. In pediatric cardiac surgery, patients require continuous monitoring for intraoperative fluid balance. The TFCd0%, which correlated well with body weight gain, body weight gain%, and intraoperative fluid balance, may be more useful indicator than the conventional methods for fluid management.

In conclusion, TFCd0%, derived using a bioreactance technique, correlated well with body weight gain, body weight gain%, and intraoperative fluid balance. It is therefore a useful indicator in the intraoperative fluid management of pediatric patients undergoing a Fontan procedure.

## Figures and Tables

**Table 1:** Patient demographic characteristics.

Subjects	(*n* = 21)
Age (months)	31 ± 5
Gender (M/F)	8/13
Height (cm)	87.8 ± 3.0
Weight (kg)	12.4 ± 1.3
Body mass index	16.1 ± 1.2

Values are presented as mean ± SD or numbers of patients.

**Table 2:** Comparisons of parameters between *T*0 and *T*1.

	*T*0	*T*1	*p* value
MAP (mmHg)	65.5 ± 8.5	60.3 ± 10.0	0.078
HR (beats per min)	103.1 ± 13.4	124.1 ± 14.9^*∗*^	<0.001
CVP (mmHg)	10.6 ± 2.2	14.4 ± 3.2^*∗*^	<0.001
CO (L/min)	1.0 ± 0.4	0.9 ± 0.4	0.235
CI (L/min/m^2^)	1.9 ± 0.6	1.6 ± 0.7	0.181
TFC (1/Ω)	55.2 ± 24.7	69.3 ± 25.8^*∗*^	<0.001
SVV (%)	13.3 ± 4.3	15.6 ± 2.9^*∗*^	0.009
Body weight (kg)	12.5 ± 1.4	13.2 ± 1.5^*∗*^	<0.001

Values are presented as means ± SD; ^*∗*^*p*  <0.05 versus *T*0. MAP, mean arterial blood pressure; HR, heart rate; CVP, central venous pressure; CO, cardiac output; CI, cardiac index; TFC, thoracic fluid content; SVV, stroke volume variation; *T*0, after anesthesia induction but before surgical incision; *T*1, just before departure from the operating room to the intensive care unit.

**Table 3:** Perioperative parameters.

Subject	(*n* = 21)
Op time (min)	223 ± 39
Anesthesia time (min)	300 ± 53
Weight gain (g)	690.0 ± 307.1
Weight gain% (%)	5.5 ± 2.2
Intake/output (ml)	680.4 ± 332.2
Change of CVP value (mmHg)	3.8 ± 3.3
ΔTFC (1/Ω)	14.1 ± 5.5
TFCd0% at discharge (%)	30.1 ± 13.3
SVV at discharge (%)	15.6 ± 2.9

Values are presented as means ± SD. Op, operation; Intake/output, intraoperative fluid balance; ΔTFC, change of thoracic fluid content compared with baseline value; TFCd0%, percentage of change compared with thoracic fluid content at baseline; CVP, central venous pressure; SVV, stroke volume variation.

**Table 4:** Comparisons of parameters between low TFCd0% group versus high TFCd0% group.

	Low TFCd0% group	High TFCd0% group	*p*value
Body weight gain (g)	550.9 ± 237.9	843.0 ± 311.6^*∗*^	0.025
Body weight gain% (%)	4.5 ± 1.7	6.6 ± 2.2^*∗*^	0.023
Intraop. fluid balance (ml)	537.3 ± 313.9	837.8 ± 288.3^*∗*^	0.035
Absolute CVP value (mmHg)	13.5 ± 3.3	15.3 ± 3.0	0.22
Change of CVP value (mmHg)	3.5 ± 3.6	4.0 ± 3.0	0.758
ΔTFC (1/Ω)	11.2 ± 5.3	17.2 ± 4.0^*∗*^	0.009

Values are presented as means ± SD; ^*∗*^*p*  <0.05 versus low TFCd0% group. Intraop, intraoperative; CVP, central venous pressure; ΔTFC, difference in thoracic fluid content.

## Abbreviations

CPB: Cardiopulmonary bypass
CVP: Central venous pressure
PCWP: Pulmonary capillary wedge pressure
BCPS: Bidirectional cavopulmonary shunt
TFC: Thoracic fluid content
TFCd0%: Percent change of thoracic fluid content compared to the baseline
NICOM: Noninvasive cardiac output monitoring
MAP: Mean arterial pressure
CO: Cardiac output
CI: Cardiac index
SV: Stroke volume
SVV: Stroke volume variation
ICU: Intensive care unit
ΔTFC: The difference in thoracic fluid content
PAP: Pulmonary artery pressure.
